# A Pipe Line for Natural Brine

**Published:** 1881-10

**Authors:** 


### A PIPE LINE FOR NATURAL BRINE.

In a report on the saline interests of Michigan, Dr. S. S. Garrigues, State Salt Inspector, mentions the construction of a pipe line for conveying brine from East Tawas to Oscoda, to be finished this fall. The pipe is of nine inch bore, of the Wyckoff patent, manufactured by the Michigan Pipe Company, and is expected to deliver brine enough to make at least 1,000 barrels of salt a day. The pipe will be laid three feet underground, and will be twelve and a half miles long. The difference of level between the two points is not given. The pumping works, consisting of two tubular boilers, 6x16, and two powerful engines, with necessary machinery, will be at East Tawas. The salt rock at that place is 196 feet thick, and its brine produces salt second to none in the State. The wells there yield on an average 200 barrels a day, while at Oscoda the yield is but about thirty barrels, and the wells do not furnish a supply equal to the capacity of the works.—*Scientific American.*

The Camphor Tree has been planted near Los Angeles, California, with every prospect of success. Why not also the cork oak, which is proved by a tree or two to be perfectly adapted to the climate, there being a good sized one in the town of Santa Barbara. Is it too slow for American enterprise?—*Gardener's Monthly.*

